# TADF Parameters in the Solid State: An Easy Way to Draw Wrong Conclusions

**DOI:** 10.1021/acs.jpca.0c10391

**Published:** 2021-02-12

**Authors:** Tomas Serevičius, Rokas Skaisgiris, Gediminas Kreiza, Jelena Dodonova, Karolis Kazlauskas, Edvinas Orentas, Sigitas Tumkevičius, Saulius Juršėnas

**Affiliations:** † Institute of Photonics and Nanotechnology, Vilnius University, Sauletekio 3, LT-10257 Vilnius, Lithuania; ‡ Institute of Chemistry, Vilnius University, Naugarduko 24, LT-03225 Vilnius, Lithuania

## Abstract

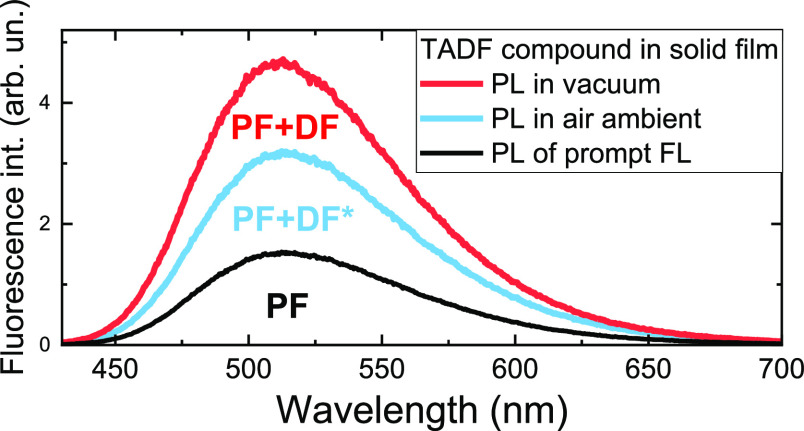

The successful development of thermally activated delayed fluorescence (TADF) OLEDs relies on advances in molecular design. To guide the molecular design toward compounds with preferable properties, special care should be taken while estimating the parameters of prompt and delayed fluorescence. Mistakes made in the initial steps of analysis may lead to completely misleading conclusions. Here we show that inaccuracies usually are introduced in the very first steps while estimating the solid-state prompt and delayed fluorescence quantum yields, resulting in an overestimation of prompt fluorescence (PF) parameters and a subsequent underestimation of the delayed emission (DF) yield and rates. As a solution to the problem, a working example of a more sophisticated analysis is provided, stressing the importance of in-depth research of emission properties in both oxygen-saturated and oxygen-free surroundings.

## Introduction

According to spin statistics, only 25% of excitons in the typical OLED device are of a singlet nature. To enhance the internal quantum efficiency of a device with singlet emitters, nonemissive triplet excitons should be employed. As a solution, the thermal activation of triplet excitons and the subsequent reverse intersystem crossing (rISC) in TADF compounds allow us to utilize nearly all of the excited states and attain efficient emission.^1−3^ To enable triplet recycling, the lowest-energy singlet and triplet states should be nearly isoenergetic.^4^ Furthermore, to ensure high TADF efficiency, a prompt fluorescence radiative decay rate (*k*_r_) should be greater than the nonradiative decay, and the rISC rate (*k*_rISC_) should exceed that of nonradiative triplet decay.^5^ Moreover, TADF OLED stability^6^ and a low external quantum yield (EQE) roll-off^7^ also rely on maximizing the *k*_r_ and *k*_rISC_ values. To optimize the material properties and later relate to device performance, fluorescence and electroluminescence yields should be estimated thoroughly. The most important parameters of prompt and delayed fluorescence (e.g., the rates of intersystem crossing (ISC) and reverse intersystem crossing), radiative and nonradiative fluorescence rates are calculated starting from the simplest ones–prompt and delayed fluorescence quantum yields (Φ_PF_ and Φ_DF_, respectively) and the corresponding fluorescence decay rates (*k*_PF_ and *k*_DF_, respectively).^5,8,9^ Prompt and delayed fluorescence quantum yields usually are estimated either by simply measuring the efficiencies under oxygen-saturated (Φ_PL_^+O_2_^) and oxygen-free (Φ_PL_^–O_2_^) ambient conditions^10,11^
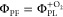
1
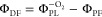
2or by deconstructing the fluorescence decay transient into prompt and delayed parts by fitting the fluorescence decay with a biexponential model and later estimating emission yields as^12,13^
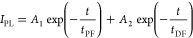
3
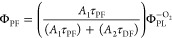
4
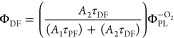
5where *A*_1_ and *A*_2_ are the fractional intensities of prompt and delayed fluorescence and τ_PF_ and τ_DF_ are the prompt and delayed fluorescence lifetimes. The first method relies on the assumption that TADF is quenched by molecular oxygen under oxygen-saturated conditions and only prompt fluorescence is observed. This is typically observed in dilute solutions, but when TADF emitters are dispersed in solid films, this is rarely the case. Dense solid surrounding efficiently prevents oxygen diffusion inside the film, when the emitter molecules close to the surface are susceptible.^14−16^ Typically, an evident part of only weakly quenched TADF still exists under +O_2_ conditions, making the direct application of eqs 1 and 2 inaccurate.^17^ Also, the unquenched part of TADF is larger for compounds with larger rISC rates since the rapid upconversion of triplet states reduces the chance of nonradiative collision with molecular oxygen,^17^ especially complicating the analysis of novel TADF materials with rapid rISC. The second method relies on the assumption that all of the delayed fluorescence is collected during the measurement. However, the TADF lifetime usually is evidently prolonged in solid films, as compared to that in solutions, due to the presence of conformational disorder.^17−20^ For compounds with less rigid molecular structure, weak delayed emission (e.g., 10^7^ times weaker than the initial intensity) can be observed even after 0.1 s,^17^ making the measurements of the TADF transient rather complicated.

In this article, we showcase an easy risk to estimate the prompt and delayed fluorescence parameters with large variation, which might eventually lead to inaccurate conclusions. We show that fluorescence decay rates may be estimated within the 1 order of magnitude error, depending on the accuracy of the initial emission parameters. Such variation of TADF rates significantly complicates the analysis and comparison of material parameters and the prediction of OLED performance. On the other hand, we show that reliable emission parameters can be obtained after the thorough analysis.

## Methods

TADF compounds were analyzed in 1 wt % PMMA (**PXZPM**, **4CzPN**), 7 wt % mCP (**tCz-ND**), and 3 wt % TSPO1 (**ARCPyr**) films. A larger doping concentration in mCP/TSPO1 films was used to ensure the full energy transfer from host to emitter and simultaneously prevent concentration quenching. Films were prepared by dissolving each material and host in appropriate ratios in toluene solutions and then wet-casting the solutions on quartz substrates. Time-integrated fluorescence spectra and fluorescence decay transients were measured using nanosecond YAG:Nd^3+^ laser NT 242 (Ekspla, τ = 7 ns, pulse energy 200 μJ, repetition rate 1 kHz) and time-gated iCCD camera New iStar DH340T (Andor). Fluorescence transients were obtained by exponentially increasing the delay and integration times.^21^ Fluorescence quantum yields (±5% error) were estimated using the integrated sphere method^22^ by integrating the sphere (Sphere Optics) connected to CCD spectrometer PMA-12 (Hamamatsu) via optical fiber. Solid-state samples were mounted in a closed-cycle He cryostat (Cryo Industries 204N) for all fluorescence measurements (for oxygen-saturated and oxygen-free conditions).

## Results and Discussion

**Figure 1:**
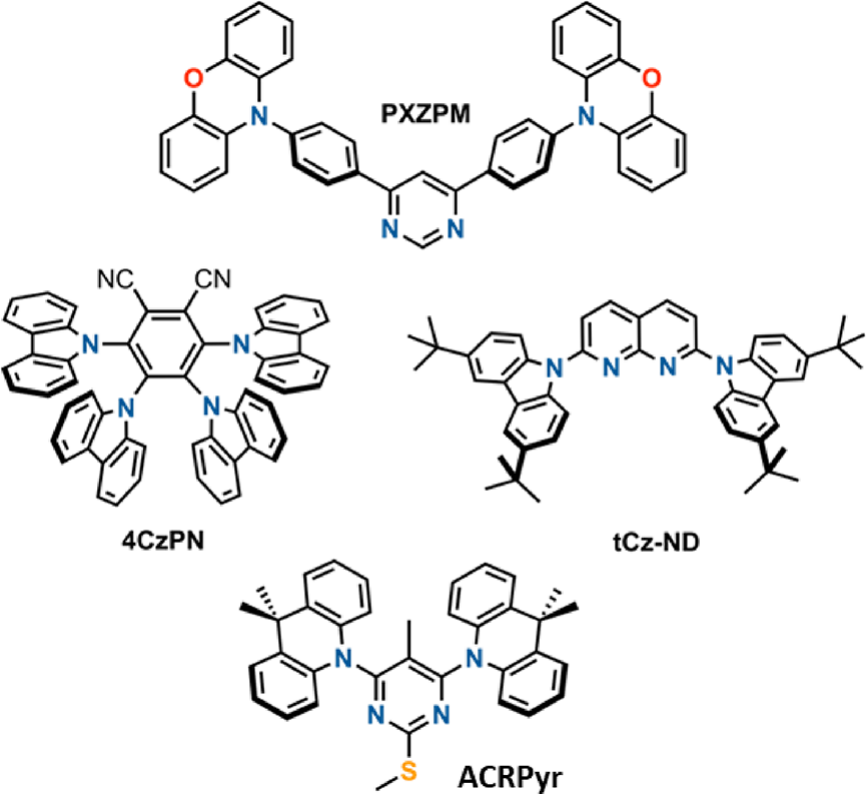
Molecular structures of compounds **PXZPM**, **4CzPN**, **tCz-ND**, and **ACRPyr**.

Four TADF compounds were analyzed (Figure 1). Extensively analyzed compound **PXZPM**(23−26) was selected as a model compound to showcase the peculiarities of Φ_PF_ and Φ_DF_. However, **PXZPM** has a rather flexible molecular core and shows evident conformational disorder.^26^ Compounds **4CzPN**,^1,17^**tCz-ND**,^27^ and **ACRPyr**,^28^ however, were selected due to the rigid molecular core and minor conformational disorder, enabling the comprehensive analysis of solid-state emission properties.

**Figure 2:**
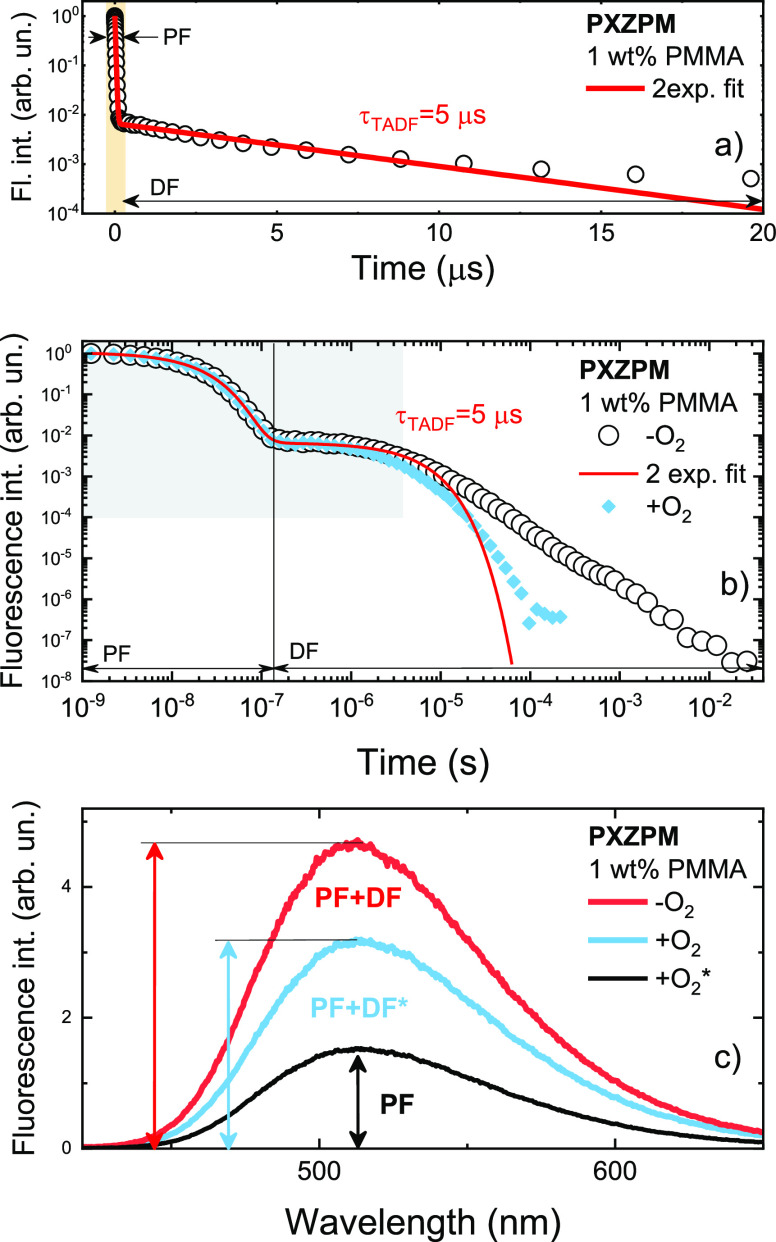
(a) Fluorescence decay transient of a 1 wt % PMMA film of **PXZPM** in a narrow intensity and temporal range under −O_2_ conditions. (b) Fluorescence decay transient of a 1 wt % PMMA film of **PXZPM** over a broad intensity and temporal range under +O_2_/–O_2_ conditions. The shaded area represents the range used in Figure 1a. (c) Fluorescence spectra of 1 wt % PMMA films of **PXZPM** under +O_2_/–O_2_ conditions. The black line represents the emission spectrum of solely prompt fluorescence, excluding the existing DF part.

**Figure 3:**
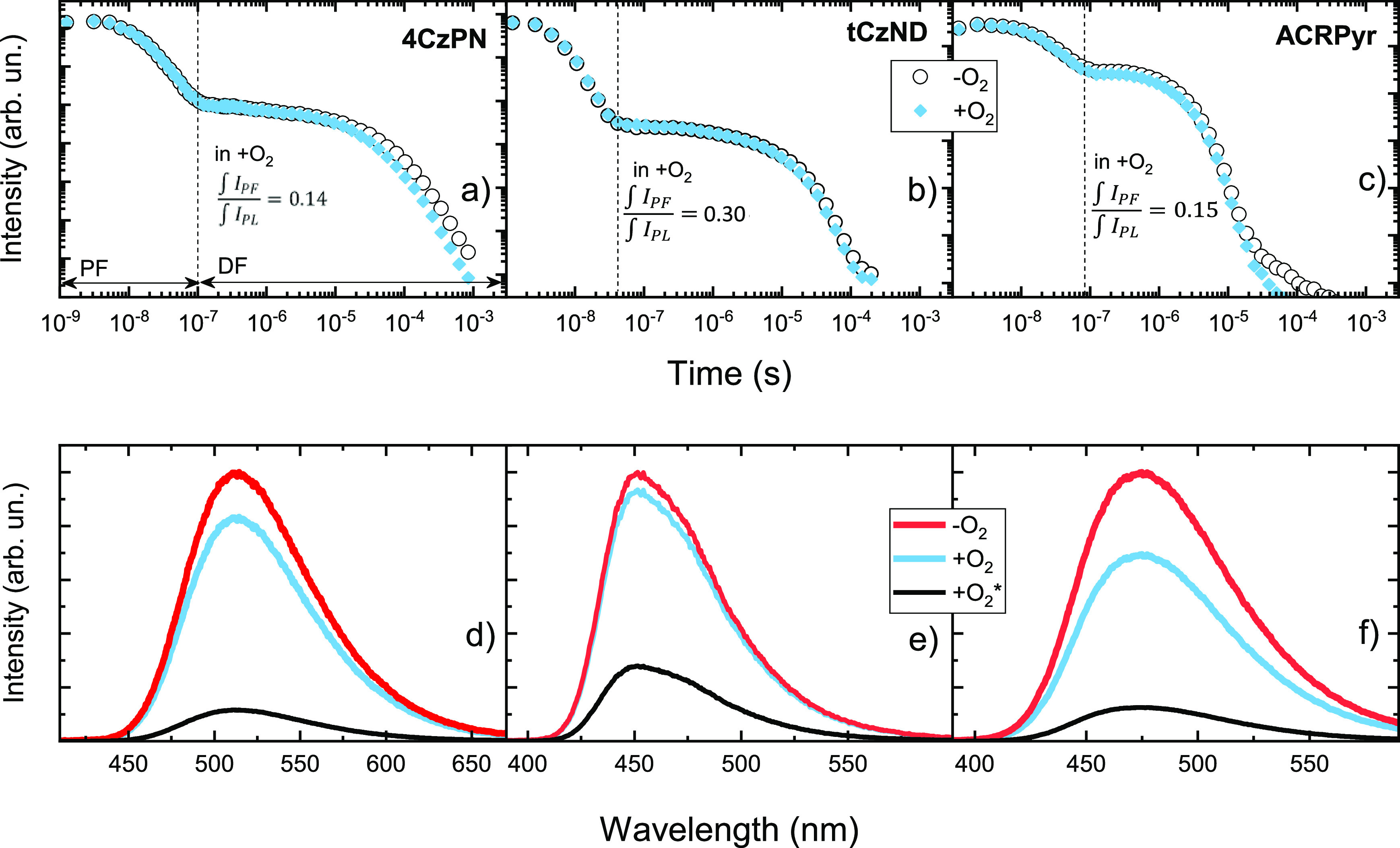
(a–c) Solid-state fluorescence decay transients of compounds **4CzPN**, **tCN-ND**, and **ACRPyr** under +O_2_ and −O_2_ conditions. The PF share in the total decay under +O_2_ conditions is shown for every compound. (d–f) Solid-state fluorescence spectra of compounds **4CzPN**, **tCN-ND**, and **ACRPyr** under +O_2_, −O_2_, and +O_2_ conditions without the DF part (+O_2_*).

Initially, phenoxazine-pyrimidine compound **PXZPM** was analyzed. Compound **PXZPM** was shown to be an efficient green TADF emitter with a fluorescence quantum yield of 1 in the mCPCN host and prompt and delayed fluorescence lifetimes of 20.2 ns and 2.56 μs, respectively, with similar parameters in the PMMA polymer host.^25^ Prompt fluorescence was shown to dominate the emission with Φ_PF_ = 0.65 and *k*_r_ = 3.22 × 10^7^ s^–1^. Fluorescence decay transients of **PXZPM** dispersed in the PMMA host at a 1 wt % doping level are shown in Figure 2a. The intensity and temporal ranges were selected to be identical to those reported in ref (25). Namely, the fluorescence intensity scale ranged from 1 to 10^–4^, while the timescale ranged from 0 to 20 μs. As we can see, the temporal profile of **PXZPM** decay is very similar to the one reported in ref (25), where the intense initial PF was observed, followed by the long-lived DF. The initial TADF decay followed a nearly single-exponential decay profile, similar to that in ref (25) with a comparable decay constant (τ_TADF_ = 5 μs). However, the situation in Figure 2a is only a small part of the big picture. Actually, the weak delayed emission of **PXZPM** is observed even up to about 20 ms, as evident from the TADF transient over a wide intensity and time range (Figure 2b). Indeed, the fractional intensity of the delayed fluorescence, according to the analysis by eqs 3–5, is clearly larger, amounting to about 73% of the total emission, which is more than twice the value stated in ref (25). Similar fractions of prompt and delayed fluorescence were also estimated by measuring the fluorescence intensity enhancement under −O_2_ conditions (Figure 2c). However, the direct use of eqs 1 and 2 would also lead to wrong conclusions. As we can see, the fluorescence intensity under ambient −O_2_ is 1.44 times larger than that under oxygen-saturated conditions. From this ratio, the DF fraction would be 59%, nearly 26% lower than the actual value. As seen in Figure 2b, a considerable part of TADF still exists under oxygen-saturated conditions, amounting to about 52% of the total emission under +O_2_ conditions. Therefore, **PXZPM** actually yields Φ_PF_ of 0.25 and Φ_DF_ of 0.67 in the PMMA film, together with a radiative decay rate of 1.29 × 10^7^ s^–1^ (*k*_r_ = Φ_PF_ × *k*_PF_), almost the same as in toluene.^26^ However, as the delayed emission, shown in Figure 2b, was clearly multiexponential due to the evident conformational disorder, it was impossible to estimate the exact TADF lifetime and compare the solid-state TADF parameters.^26^ For this purpose, three TADF emitters with rigid molecular structure and nearly single-exponential TADF decay, namely, **4CzPN**, **tCz-ND**, and **ACRPyr**, were analyzed (Figures 1 and 3).

**Figure 4:**
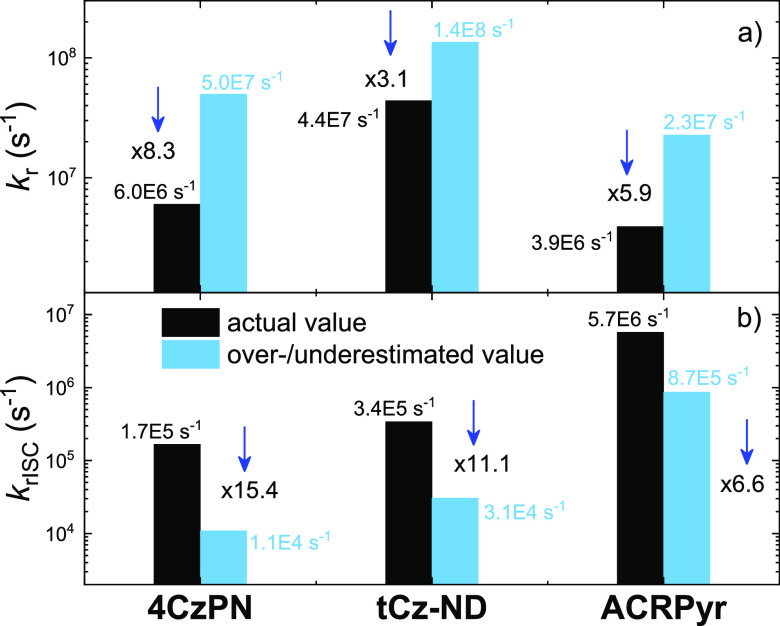
(a) Fluorescence radiative decay and (b) reverse intersystem crossing rates for compounds **4CzPN**, **tCN-ND**, and **ACRPyr**. Accurate values are shown as black bars, and inaccurate values are shown as blue bars. The numbers close to the arrows denote the ratio between both values. *k*_rISC_ was calculated according to the models used in the initial reports. *k*_rISC_ of **4CzPN** was calculated according to Kreiza et al.^8^

All three compounds showed intense and rather short-lived delayed fluorescence (τ_TADF_ ranged from 1.76 to 46 μs). This rapid delayed fluorescence was weakly quenched under +O_2_ conditions (Figure 3a–c), leading to a minor PL intensity difference under oxygen-saturated and oxygen-deficient conditions (Figure 3d–f). The direct use of eqs 1 and 2 would give Φ_PF_ for all three TADF compounds in the range from 0.44 to 0.7 (0.44, 0.6, and 0.7 for **ACRPyr**, **4CzPN**, and **tCN-ND**, respectively). However, as we can see from Figure 3a–c, the PF share (η_PF_ = ∫*I*_PF_/∫*I*_PL_) in the total emission under +O_2_ conditions was only 0.14–0.30, leading to a remarkably lower real Φ_PF_ of 0.07–0.23 (0.07, 0.09, and 0.23 for **ACRPyr**, **4CzPN**, and **tCN-ND**, respectively, equation Φ_PF_ = Φ_PL_^+2^η_PF_) and a remarkably larger real Φ_DF_ of 0.53–0.63 (0.59, 0.64, and 0.53 for **ACRPyr**, **4CzPN**, and **tCN-ND**, respectively; eq 2). Such variation in Φ_PF_ and Φ_DF_ values leads to very large discrepancies between accurate and inaccurate TADF parameters. This is shown in Figure 4, where the radiative fluorescence decay and rISC rates are compared. Both *k*_r_ and *k*_rISC_ were showcased as both strongly depending on the emission yield, and both rates are used for the estimation of other major fluorescence parameters.^5,8^

As shown, accurate *k*_r_ ranged from 3.9 × 10^6^ s^–1^ (**ACRPyr**) to 44.2 × 10^6^ s^–1^ (**tCz-ND**). Such a high *k*_r_ for **tCN-ND** was in line with the rapid PF decay and high oscillator strength of the S_0_ → S_1_ transition.^27^ However, the rapid *k*_r_ with values exceeding 10^7^, 1 order of magnitude larger than the accurate ones, could be derived if the overestimated Φ_PF_ was used. In this case, *k*_r_ ranged from 22.7 (**ACRPyr**) to the remarkable 135 × 10^6^ s^–1^ for **tCz-ND**. *k*_r_ values of >10^8^ s^–1^ are typical for organic lasing materials with strong LE emission^29^ and are hardly likely for CT-based TADF compounds.^30^ On the contrary, the rISC rate was underestimated even more. The actual *k*_rISC_ ranged from 0.2 × 10^6^ s^–1^ for **4CzPN** to 5.7 × 10^6^ s^–1^ for **ACRPyr**. When the enlarged Φ_PF_ was used, *k*_rISC_ decreased down to 0.011 × 10^6^ s^–1^ (**4CzPN**) to 0.9 × 10^6^ s^–1^ (**ACRPyr**). Clearly, such a deviation in the fluorescence parameters by up to 1 order of magnitude complicates the material optimization and may provide wrong guidelines, as the impact of delayed fluorescence is evidently underestimated. Somewhat similar results should be obtained if only the initial and intense delayed fluorescence is accounted for in the fluorescence transients, as shown in Figure 2d–f. Therefore, to avoid such tremendous errors in solid-state TADF parameters, great care should be taken. For instance, the existing DF part should be eliminated from Φ_PF_ under +O_2_ conditions. Concomitantly, the TADF transients should be measured over large intensity and temporal ranges, including the weak DF at the largest delays.^21,31^

## Conclusions

We have shown that solid-state TADF parameters can be estimated with high inaccuracy. The specific solid-state surrounding prevents the full delayed fluorescence quenching in ambient air; therefore, it is critically important to exclude the remaining DF part in order to get the correct prompt and delayed fluorescence quantum yields according to eqs 1 and 2. On the other hand, the conformational disorder existing in the solid state usually remarkably extends the delayed fluorescence lifetime, when the latest weak delayed fluorescence is difficult but critical to assess. Failing to do that, prompt and delayed fluorescence parameters, according to eqs 1–5, can be estimated within 1 order of magnitude error, which is highly unfavorable for material and device optimization.
